# Longitudinal BMI-for-Age Trajectories Among Mexican School-Aged Children, 2015–2019

**DOI:** 10.3390/nu18213532

**Published:** 2026-10-27

**Authors:** Abelardo Cesar Avila-Curiel, Teresa Shamah-Levy, Elsa Berenice Gaona-Pineda, Marco Antonio Avila-Arcos, Marti Yareli Del Monte-Vega, Lucia Cuevas-Nasu, Ignacio Méndez Gómez-Humarán

**Affiliations:** 1 Department of Applied Nutrition and Nutrition Education, National Institute of Medical Sciences and Nutrition Salvador Zubiran, Mexico City PC 14000, Mexico; abelardoavila@yahoo.com (A.C.A.-C.); marti.delmonte@incmnsz.mx (M.Y.D.M.-V.); 2 Center for Evaluation and Surveys Research, National Institute of Public Health, Cuernavaca PC 62100, Mexico; elsa.gaona@insp.mx (E.B.G.-P.); marco.avila@insp.mx (M.A.A.-A.); lcuevas@insp.mx (L.C.-N.); 3 Center for Research in Mathematics-Aguascalientes Campus, Aguascalientes PC 20200, Mexico; imendez@cimat.mx

**Keywords:** school-aged children, obesity, overweight, nutritional surveillance, Mexico

## Abstract

Background/Objectives: Childhood body mass index (BMI) is dynamic, and cross-sectional estimates do not capture within-child changes over time. This study aimed to characterize longitudinal BMI-for-age trajectories among Mexican school-aged children and examine differences according to individual and school-context characteristics. Methods: We analyzed 463,244 children aged 5–12 years with valid anthropometric measurements over four consecutive school years (2015–2016 to 2018–2019). BMI-for-age z-scores (BAZ) were calculated using the WHO 2007 growth reference. Longitudinal patterns were identified using latent class mixed models. Multinomial logistic regression estimated the proportions of children belonging to each trajectory group, and population-averaged generalized estimating equations (GEE) examined overweight/obesity across school grades and trajectory groups. Results: Four trajectories were identified, broadly corresponding to low-BAZ, mid-range BAZ, elevated BAZ, and very-high BAZ trajectories, comprising 13.08%, 43.68%, 32.01%, and 11.23% of children, respectively. Trajectory-group membership probabilities differed by sex and school-context characteristics. Girls had a higher proportion in the mid-range BAZ trajectory than boys (45.7 vs. 41.7%) but a lower one in the very-high BAZ trajectory (8.4 vs. 14.0%). Elevated BAZ and very-high BAZ trajectory proportions increased as municipal marginalization decreased. In the GEE model, overweight/obesity increased across school grades, particularly in the elevated BAZ trajectory (grade-by-trajectory interaction (OR = 1.33; *p* < 0.001); girls had lower (OR = 0.94; *p* < 0.001) and children attending urban schools higher (OR = 1.1; *p* < 0.001) overweight/obesity. Conclusions: Mexican school-aged children exhibited heterogeneous longitudinal BMI-for-age patterns. Individually linked repeated anthropometric measurements provide information on within-child BMI patterns that complement cross-sectional prevalence estimates and may improve the characterization of childhood nutritional status across school and geographic contexts.

## 1. Introduction

Childhood overweight and obesity constitute a major global public health challenge. A systematic review of 2033 studies from 154 countries or regions estimated that 22.2% of children and adolescents had overweight or obesity [1]. Similarly, a pooled analysis of population-based studies documented a marked global increase in obesity between 1990 and 2022 [2]. In 2022, the World Health Organization estimated that more than 390 million children and adolescents aged 5–19 years had overweight, including approximately 160 million living with obesity [3]. Childhood obesity is associated with cardiometabolic, musculoskeletal, respiratory, and psychosocial complications and is more likely to persist into adulthood [4].

Mexico faces a particularly high burden of childhood overweight and obesity. Data from the National Health and Nutrition Survey (Ensanut acronym in Spanish: Encuesta Nacional de Salud y Nutrición) 2020–2022 showed prevalences of 19.2% for overweight and 18.1% for obesity among children aged 5–11 years [5]. Estimates from Ensanut 2020–2023 similarly indicated that 36.5% of school-aged children had overweight or obesity [6]. Childhood overweight and obesity in Mexico occur within a complex context of individual, family, social, and environmental determinants [7]. Dietary patterns characterized by high consumption of added sugars and low consumption of fruits and vegetables have also been documented among Mexican school-aged children [8].

Previous analyses of the Mexican National Registry of Weight and Height (Registro Nacional de Peso y Talla, RNPT in Spanish) provided important evidence on the distribution and temporal patterns of childhood overweight and obesity. An analysis including approximately 6.5 million students from 59,724 public elementary schools compared overweight and obesity across four school periods from 2015 to 2018; the study identified higher odds of overweight and obesity among boys and children attending schools in urban and less marginalized areas, as well as increasing obesity in the southern region [9]. However, its comparative cross-sectional design did not link repeated measurements from the same children; therefore, it could not characterize individual weight trajectories or transitions between nutritional status.

Cross-sectional surveys and surveillance systems are essential for estimating the prevalence of overweight and obesity and monitoring population-level trends; however, they cannot capture within-child changes in BMI-for-age over time. Longitudinal studies have identified heterogeneous childhood weight trajectories, including persistence of overweight and obesity, progression from overweight to obesity, and transitions toward a healthier weight status [10,11,12,13,14]. Importantly, relatively stable population prevalence may coexist with substantial individual changes in nutritional status. Characterizing longitudinal BMI-for-age trajectories may therefore provide information that cannot be obtained from repeated cross-sectional estimates alone. School age represents an important period for understanding the development and persistence of excess weight. Schools also constitute an important setting for nutrition education, physical activity, health services, and improvements in food environments. Multicomponent school-based interventions can contribute to healthier practices and weight-related outcomes, although their effectiveness varies according to their components, duration, and implementation context [15]. Identifying when unfavorable BMI-for-age trajectories emerge or persist during the school years may therefore contribute to identifying relevant opportunities for prevention.

The availability of standardized weight and height measurements collected from the same children during four consecutive school years provides an opportunity to extend the previous analyses of the National Registry of Weight and Height by examining individual BMI-for-age trajectories. Characterizing longitudinal BMI-for-age patterns may provide insights into heterogeneity of BMI development during the elementary school years and help identify groups that may benefit from preventive interventions.

Therefore, this study aimed to identify and characterize longitudinal BMI-for-age z-score trajectories among Mexican school-aged children with four consecutive annual anthropometric measurements from 2015–2016 to 2018–2019, and to examine differences in trajectory-group membership according to individual and school-context characteristics.

## 2. Materials and Methods

### 2.1. Study Design and Population

This retrospective longitudinal study used data from the Mexican National Registry of Weight and Height (RNPT). The RNPT is a nationwide school-based nutritional surveillance system previously described in detail, México, 2016 [16]. The available dataset comprised 1,852,976 valid weight and height measurements from 463,244 children attending 17,372 schools across Mexico, collected during four consecutive school years: 2015–2016, 2016–2017, 2017–2018, and 2018–2019.

Individual records were longitudinally linked across the four school years using the Clave Única de Registro de Población (CURP), Mexico’s unique population identification code. For confidentiality and data protection, the CURP was encrypted and anonymized and used exclusively as an individual linkage identifier to identify repeated measurements belonging to the same child across the four assessment periods. The analytical cohort was restricted to children aged 5–12 years at baseline (2015–2016) who had one valid anthropometric measurement in each of the four consecutive school years. The age eligibility criterion was applied exclusively at baseline, and children were subsequently followed across the remaining three school years. Thus, each child included in the analytical cohort contributed four repeated anthropometric observations, enabling the evaluation of within-child changes in BMI-for-age and nutritional status over time.

The number of potentially eligible children excluded because of incomplete longitudinal follow-up could not be reconstructed from the available analytical dataset. Therefore, the proportion of children excluded for this reason could not be quantified.

Children enrolled in fifth or sixth grade at baseline (2015–2016) were also included if they had four consecutive annual anthropometric measurements.

### 2.2. Data Source

The RNPT was coordinated by the Department of Applied Nutrition and Nutritional Education of the National Institute of Medical Sciences and Nutrition Salvador Zubirán (INCMNSZ) and included anthropometric information collected from children attending public and private elementary schools in Mexico [16]. The RNPT was a study in which participation by schools across the country was voluntary; therefore, it is not considered representative of Mexico’s elementary schools. Weight and height measurements were obtained by personnel from the health and education sectors according to the procedures established by the RNPT [9].

Anthropometric and identification data were recorded through the School-aged children Nutritional Surveillance System (Sistema Informático de Vigilancia Nutricional en Escolares, SIVNE), an electronic information system developed to capture, store, organize, georeference, and process anthropometric data from school-aged children. The RNPT data collection procedures, instruments, and information system have been described previously [16] and using internationally validated methodologies [17].

### 2.3. Anthropometric Measurements and Nutritional Status

Due to the large size of the dataset, SQL server was used to store and process the data, and tSQL scripts based on WHO guidelines were used to obtain age, BMI, and Score-Z values: age in months was computed as the day difference between birth date and measure date divided by 30.4375, body mass index (BMI) was calculated as weight in kilograms divided by height in meters squared (kg/m^2^), and age-and sex-specific BMI-for-age z-scores (BAZ) were calculated using the 2007 World Health Organization (WHO) growth reference for children and adolescents aged 5–19 years using the LMS tables and their respective formulas [18,19]. Nutritional status at each of the four assessments was classified according to the WHO criteria as thinness (BAZ < −2 SD), normal weight (BAZ ≥ −2 to ≤+1 SD), overweight (BAZ > +1 to ≤+2 SD), and obesity (BAZ > +2 SD) [19]. The BMI-for-age z-scores calculated using SQL Server were also validated against those obtained using WHO AnthroPlus software [20].

Consequently, each child had four sequential BMI-for-age measurements and four corresponding nutritional status classifications, one for each school year. Biologically implausible BMI-for-age z-scores (BAZ < −5 or >+5 SD) were excluded from the analysis according to the WHO AnthroPlus criteria [20], considering that measurements were performed annually; all changes were considered plausible as long as the z-score values remained within this valid interval.

Personnel responsible for anthropometric assessments received training and standardization in weight and height measurement procedures, including the calibration and zeroing of weighing scales and verification of the proper functioning of stadiometers.

Duplicate records were identified using the Unique Population Registry Code (CURP) and school year.

### 2.4. Study Covariables

Individual-level variables available in the RNPT included sex and age at the time of each anthropometric measurement [16]. School grade was recorded from first through sixth grade.

School-level and contextual characteristics included type of school, urban or rural setting, and degree of marginalization. The type of school was classified as indigenous or school from the Mexican National Council for Educational Development (CONAFE by its acronym in Spanish), public school, and private school. Urban and rural settings were classified according to the 2020 National Population and Housing Census [21]. Municipal marginalization was determined using the 2020 Marginalization Index published by the National Population Council (CONAPO) [22]. Both contextual classifications were applied retrospectively to schools included in the 2015–2019 cohort based on their geographic location. These variables were treated as contextual characteristics rather than time-varying measures.

### 2.5. Statistical Analysis

The analytical cohort included 463,244 children with four consecutive annual anthropometric measurements between the 2015–2016 and 2018–2019 school years. 

Descriptive analyses were conducted to characterize the study population at the baseline (2015–2016). Categorical variables were summarized using frequencies and percentages.

For statistical analysis, group-based trajectory modeling (GBTM) was used to identify distinct longitudinal patterns in the standardized body mass index (IMCZ) from 2015 to 2018 [23]. A latent class mixed model (LCMM) was fitted to the four repeated measurements, specifying class-specific quadratic trajectories that allowed the intercept and linear and quadratic time terms to vary across latent groups [24]. School grade at baseline (2015–2016) was included as a covariate for the multinomial class-membership model to assess differences in the probability of belonging to each latent trajectory group.

Given the exceptionally large sample size (463,244 children and 1,852,976 observations), the computational requirements of fitting models with multiple latent classes made the use of multiple random starting values impractical. Therefore, candidate models with two to six latent classes were fitted using a robust 10% subsample, with the one-class quadratic model used to provide the initial parameter values for all candidate models (Supplementary Table S1). As the one-class model was used to provide the initial parameter values for all candidate models, this strategy ensured a consistent initialization procedure across candidate models, although it did not guarantee that the global maximum of the likelihood was reached. Model selection was based on a combination of statistical criteria and comparative model fit across the candidate solutions. The corresponding fit statistics are presented in Supplementary Table S1 and provide additional evidence supporting the selection of the four-class solution.

The four-class model was subsequently fitted to the full sample, retaining the statistical criteria and interpretability that supported its selection in the subsample. Specifically, all classes achieved mean posterior membership probabilities above 0.85, exceeding the minimum threshold of 0.70, while 82.41–85.34% of individuals in each class had posterior probabilities greater than 0.70. Additionally, all classes contained a substantial proportion of the sample, ranging from 11.23% to 43.68%, avoiding classes with very small membership. Together, these criteria supported adequate classification, certainty, and a meaningful representation of the population across the four trajectories. The model syntax is available in Supplementary Material S1.

A multinomial logistic regression model was used to characterize the trajectory groups identified by the GBTM according to sex, school type, urban/rural setting, and degree of municipal marginalization. The distribution of observed cases across trajectory groups was described using proportions, and their corresponding 95% confidence intervals were from the fitted multinomial model, which estimated the distribution of observed cases across trajectory groups according to the selected sociodemographic characteristics.

Following the group-based trajectory modeling (GBTM) analysis, a population-aged model based on generalized estimating equations (GEE) was used to characterize the trends in overweight and obesity prevalence represented by the four trajectory groups and examine their associations with school grade and other relevant individual and contextual factors. A binomial distribution with a logit link was specified to model the prevalence of overweight and obesity, using GEE with an exchangeable correlation structure specified to account for repeated observations. An interaction between trajectory group and school grade was included to assess whether prevalence trends across school grades differed according to trajectory group membership. Associations were quantified using odds ratios (ORs) and their corresponding 95% confidence intervals.

The population-averaged model was conducted as a secondary descriptive analysis to characterize differences in overweight and obesity prevalence across school grades by trajectory group. Because both trajectory membership and nutritional status were derived from the same repeated BMI-for-age measurements, these differences do not constitute an independent validation of the identified trajectories.

Statistical analyses were performed using the Hierarchical Linear Mixed-Effects “hlme” procedure in the Latent Class Mixed Models through the “lcmm” package; the multinomial logistic model was fitted using the “multinom” procedure from the “nnet” package; and the population-averaged model was performed using the “geeglm” procedure in the “geepack” package; all for R (Version 4.6.1) [25].

## 3. Results

### 3.1. Baseline Sample Characteristics

At baseline (2015–2016), 50.5% of the participants were boys. Most children were aged 6–8 years (89.1%), with 32.5% aged 7 years and 31.9% aged 8 years. Most children were enrolled in the first three grades of elementary school (Table 1).

Regarding school types, 87.6% attended federal public schools, 10.6% went to private schools, and 1.8% studied in indigenous or CONAFE schools. Most children attended schools located in urban localities (86.9%), and 84.7% attended schools in municipalities classified as having very low marginalization. 

At baseline, 62.0% of children were classified as having normal weight, 18.4% as having overweight, 15.7% as having obesity, and 3.9% as having thinness according to BMI-for-age z-scores.

### 3.2. Evolution of BMI z-Score Trajectories

**Figure 1:**
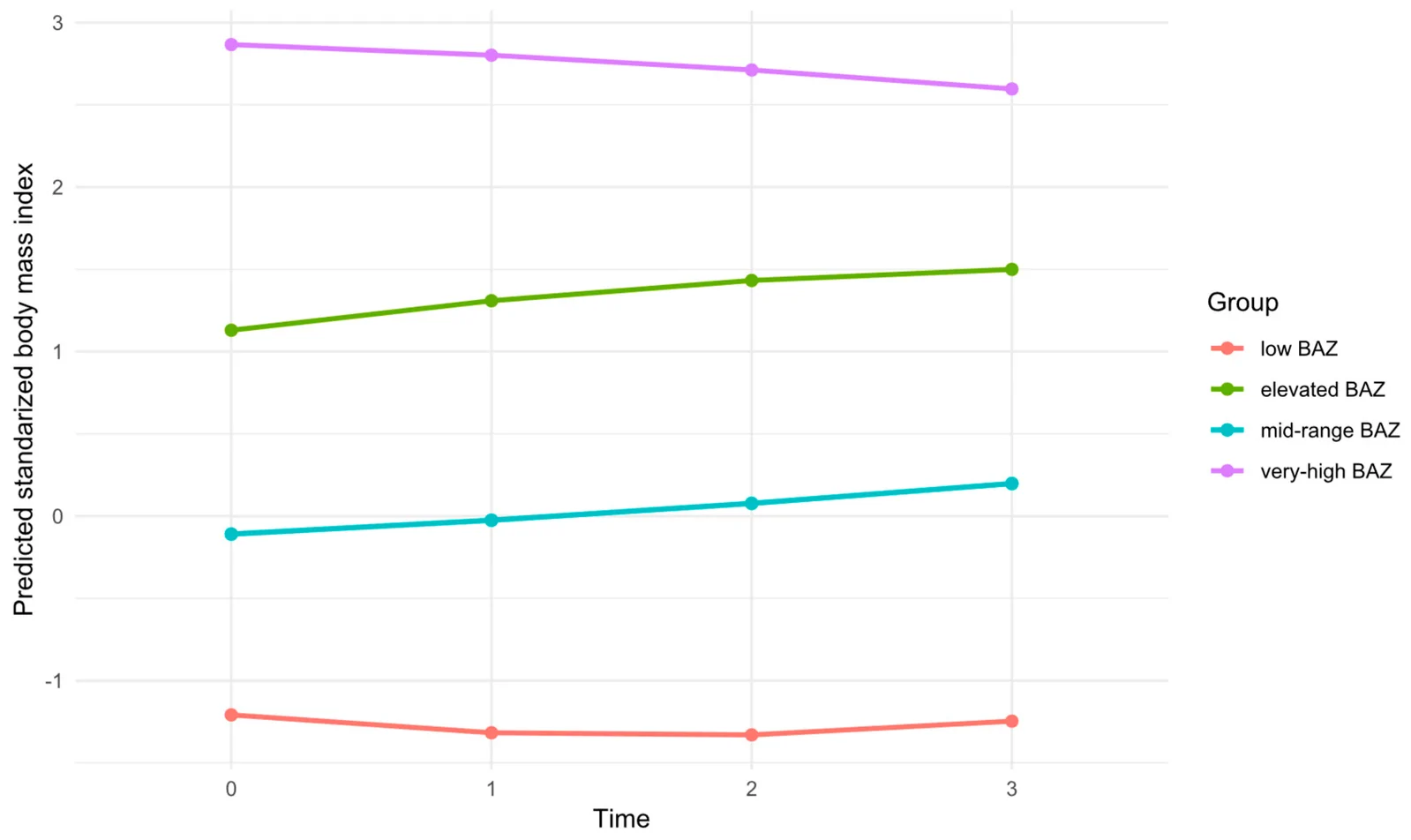
Model-estimated BMI-for-age z-score trajectories across four consecutive school years.

Four distinct longitudinal trajectory groups were identified across the four consecutive school years (Figure 1). The selected model showed good classification performance, with mean posterior probabilities of 0.872, 0.868, 0.857, and 0.890 for Groups 1–4, low-BMI-for-age z-score (BAZ), mid-range BAZ, elevated BAZ, and very-high BAZ trajectories, respectively. More than 82% of the children assigned to each trajectory group had posterior membership probabilities greater than 0.70. The resulting trajectory groups comprised 13.08%, 43.68%, 32.01%, and 11.23% of the sample, respectively.

### 3.3. Sociodemographic Characterization of BMI-for-Age Trajectory Groups

Distribution of trajectory group membership differed according to sex and school characteristics (Table 2). Among boys, the proportional distribution in groups was the lowest at 12.3% (95% CI: 12.2–12.5) for low BAZ, the highest at 41.7% (95% CI: 41.5–41.9) for mid-range BAZ, 32% (95% CI: 31.8–32.1) for elevated BAZ and 14.0% (95% CI: 13.9–14.2) for very-high BAZ, respectively. Among girls, the corresponding proportions were 13.8% (95% CI: 13.7–14) for low BAZ, 45.7% (95% CI: 45.5–45.9) for mid-range BAZ, 32.1% (95% CI: 31.9–32.2) for elevated BAZ, and 8.4% (95% CI: 8.3–8.5) for very-high BAZ. 

Differences were also observed according to the type of school. Children attending Indigenous or CONAFE schools had a proportional distribution of 15.6% for low BAZ, 58.7% for mid-range BAZ, 23% for elevated BAZ, and 2.7% for very-high BAZ. The corresponding distributions were 13.2%, 43.6%, 31.9%, and 11.3% among children attending public schools and 12%, 41.6%, 34.3%, and 12.2% among those attending private schools, respectively. 

Children attending rural schools had a higher proportion of belonging to the low-BAZ trajectory (14.7%, 95% CI: 14.4–15) than those attending urban schools (12.8%; 95% CI: 12.7–12.9). The proportional distribution in the mid-range BAZ trajectory was higher in rural schools (49.6%; 95% CI: 49.2–50) than in urban schools (42.8%; 95% CI: 42.6–42.9). Conversely, the proportional distribution in the elevated BAZ trajectory was lower among children attending rural schools (27.8%; 95% CI: 27.4–28.1) than among those attending urban schools (32.6%; 95% CI: 32.5–32.8). The proportional distribution in the very-high BAZ trajectory was lower in rural schools (7.9%; 95% CI: 7.6–8.1) than in urban schools (11.7%; 95% CI: 11.6–11.8).

Differences in trajectory-group distributions were observed across levels of municipal marginalization. The children’s distribution in the low-BAZ trajectory decreased from 14.5% (95% CI: 13.5–15.5) among children attending schools in municipalities with very high marginalization to 12.9% (95% CI: 12.8–13) among those in municipalities with very low marginalization. The distribution in the elevated BAZ trajectory increased from 20.7% (95% CI: 19.5–21.8) to 32.7% (95% CI: 32.5–32.8), whereas the proportional distribution in the mid-range BAZ trajectory decreased from 61.6% (95% CI: 60.3–63) to 42.7% (95% CI: 42.5–42.9). In contrast, the proportional distribution in the very-high BAZ trajectory increased from 3.1% (95% CI: 2.6–3.6) to 11.7% (95% CI: 11.6–11.8) as municipal marginalization decreased.

### 3.4. Trends in the Prevalence of Overweight and Obesity and Its Association with Relevant Individual and Contextual Factors

**Figure 2:**
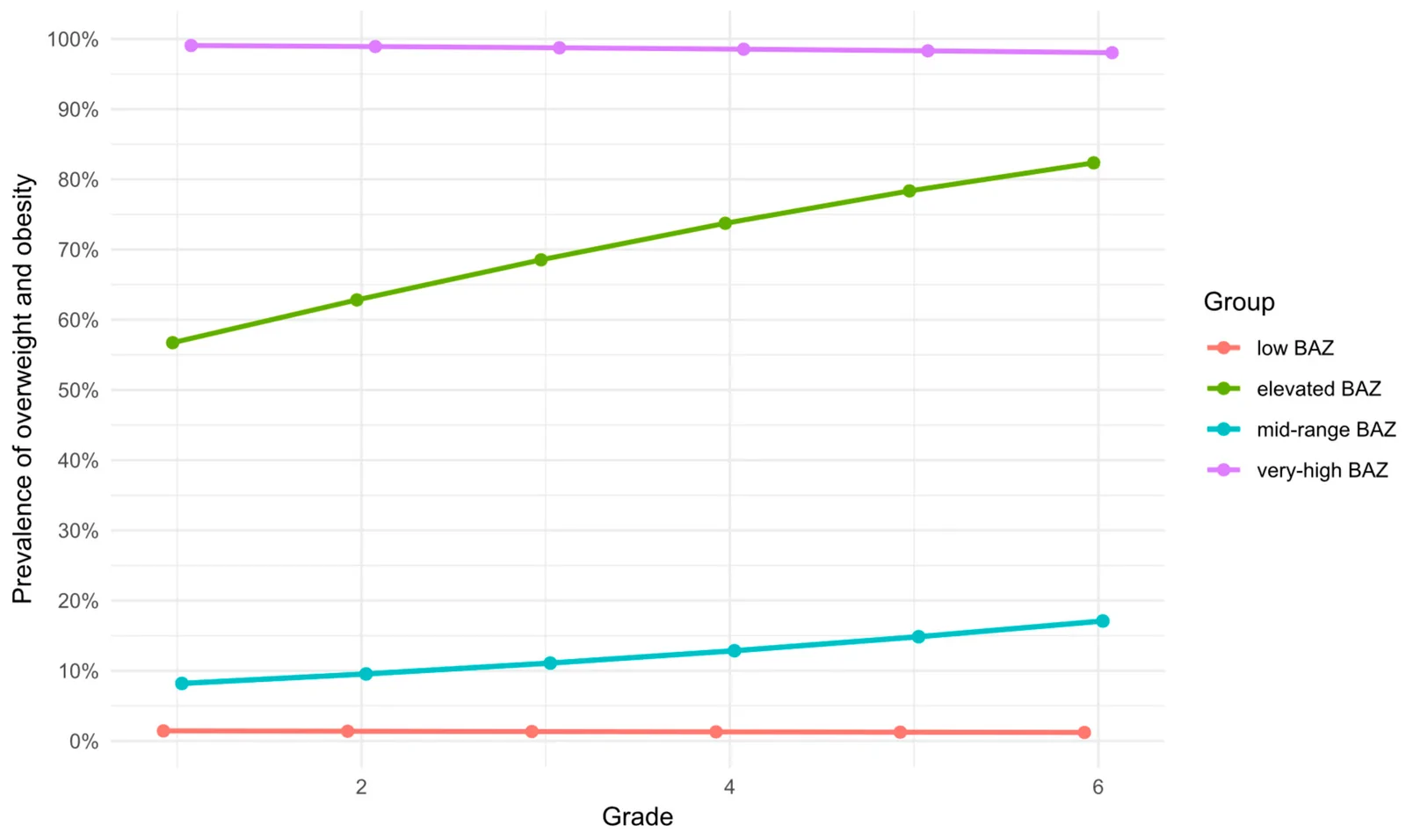
Evolution of the grouped prevalence of overweight and obesity by school grade.

The combined prevalence of overweight and obesity (Table 3) showed a markedly higher OR across school grades among the elevated BAZ group. A similar but less pronounced increase in OR was observed in the mid-range BAZ group. In contrast, the low BAZ group remained relatively stable, with a prevalence close to zero, whereas the very-high BAZ group showed a slight decrease across grades (Figure 2). 

As the level of marginalization decreased, a greater OR for the prevalence of overweight and obesity was observed; the same applied to schools in urban areas. Girls appeared to have lower odds of overweight and obesity than boys. The type of school was the only factor not associated with prevalence rates (Table 3).

## 4. Discussion

This longitudinal study of 463,244 Mexican school-aged children identified four distinct BMI-for-age trajectories across four consecutive school years, characterized as low-BAZ, mid-range BAZ, elevated BAZ, and very-high BAZ trajectories.

The trajectories showed good classification performance and revealed heterogeneous patterns of BMI development during the elementary school years. The evolution of overweight and obesity differed across trajectory groups, with an increasing pattern across school grades, particularly among children assigned to the elevated BAZ trajectory. In addition, predicted trajectory-group membership differed according to sex and school-context characteristics, including urban–rural setting and municipal marginalization. These findings extend previous cross-sectional analyses of the Mexican National Registry of Weight and Height (RNPT) by characterizing within-child BMI-for-age z-score patterns over time rather than changes derived from different groups of children at successive measurement periods.

The identification of distinct longitudinal BMI-for-age z-score patterns is consistent with previous studies that have shown substantial heterogeneity in BMI development throughout childhood. Group-based and latent-class approaches have commonly identified a limited number of distinct trajectories, although their number, shape, and relative frequency vary according to the population studied, age range, anthropometric indicator, follow-up period, and modelling strategy [12,13,26]. In a scoping review of 59 studies using group-based trajectory methods across the life course, BMI was the most frequently examined growth measure, and four was the median number of identified trajectories [26]. Similarly, Huang et al. identified four longitudinal BMI percentile trajectories—early-onset overweight or obesity, late-onset overweight or obesity, normal stable, and low-stable—using repeated anthropometric measurements from childhood through adolescence [12]. Evidence from Latin America also supports the value of longitudinal anthropometric data: a recent population-based study linking administrative and nutritional surveillance records from approximately 1.7 million Brazilian children demonstrated differences in BMI trajectories from 3 to 9 years of age, according to early life growth patterns [27]. Taken together, these studies support longitudinal approaches for characterizing heterogeneity in BMI development and within-child change over time, which cannot be captured by a single anthropometric measurement.

The longitudinal analysis further showed that the evolution of overweight/obesity across school grades differed according to trajectory group membership. The increase was most pronounced among children assigned to the elevated BAZ trajectory and was more moderate among those in the mid-range BAZ trajectory, whereas overweight/obesity remained uncommon among children assigned to the low BAZ trajectory. These findings are consistent with longitudinal evidence indicating that weight status during childhood is dynamic and that children may follow different patterns of persistence or change over time [10,12,14]. Huang et al. showed that longitudinal BMI percentile trajectories provided additional information on subsequent overweight/obesity compared with the BMI percentile measured at a single point in early life [12]. Similarly, studies based on repeated anthropometric measurements have demonstrated that the persistence and evolution of an elevated BMI vary substantially among children [10,14]. In the present study, however, trajectory membership should not be interpreted as evidence of an individual transition from one nutritional status category to another, because the latent classes represent model-derived patterns of repeated BMI-for-age z-scores rather than predefined clinical transitions.

In contrast, the prevalence of overweight/obesity showed a slight decrease across school grades among children assigned to the very-high BAZ trajectory. This finding should be interpreted cautiously and should not be considered evidence of obesity remission. The trajectory and GEE models characterize different aspects of the longitudinal data: trajectory groups summarize patterns of repeated continuous BMI-for-age z-scores, whereas the GEE evaluates a dichotomous overweight/obesity outcome across school grades. Consequently, changes in BMI for age in relation to the WHO classification thresholds may modify the probability of the dichotomous outcome without necessarily indicating a substantial change in the underlying BMI trajectory. Previous longitudinal evidence indicates that obesity during childhood can persist or remit over time, although remission becomes less frequent with increasing obesity severity [10]. In addition, simulation analyses projecting childhood BMI trajectories into adulthood have shown substantial tracking of obesity from childhood to later ages [28]. Therefore, the slight decline observed in the very-high BAZ trajectory in the present study should be interpreted as a feature of the modelled longitudinal pattern rather than as evidence of spontaneous resolution of obesity.

Sex differences were also observed in longitudinal patterns. In the adjusted GEE model, girls had lower odds of overweight/obesity than boys after accounting for school grade, trajectory-group membership, school type, municipal marginalization, and urban–rural setting. However, sex differences in the predicted distribution of trajectory-group membership did not follow a uniform pattern across latent classes. This apparent difference is not unexpected because the two analytical approaches estimate distinct features of the longitudinal data: the GEE estimates the population-averaged association between sex and the repeated dichotomous overweight/obesity outcome, whereas the trajectory model estimates the membership distribution in latent groups defined by repeated BMI-for-age z-scores. Sex-related differences in childhood BMI trajectories have also been examined in other populations. Kim et al. reported broadly similar trajectory patterns for boys and girls, although boys showed slightly higher BMI-for-age z-scores across childhood [29]. Differences between studies should be interpreted cautiously because trajectory patterns depend on the population, age range, follow-up period, and modelling approach.

Contextual differences were also observed in the longitudinal occurrence of overweight/obesity. In the adjusted GEE model, children attending schools in urban settings had higher odds of overweight/obesity than those in rural settings. A gradient was also observed across the levels of municipal marginalization, with progressively higher odds of overweight/obesity as marginalization decreased, reaching the largest estimate among children attending schools located in municipalities with very low marginalization. These GEE findings refer to repeated overweight/obesity outcomes and should be distinguished from the trajectory group membership distribution estimated using the multinomial model. This pattern is consistent with recent evidence from Mexico showing socioeconomic inequalities in childhood and adolescent overweight/obesity, including higher odds among those living in urban areas and households with greater wealth [30]. However, socioeconomic indicators are not interchangeable, and municipal marginalization in the present study represents an area-level contextual measure rather than an individual or household’s socioeconomic position. Studies from other settings have also documented associations between neighbourhood socioeconomic conditions and childhood BMI trajectories [29,31], although the direction and magnitude of these associations vary according to the population and socioeconomic measures considered. Therefore, the observed associations cannot establish whether the differences were attributable to household resources, dietary patterns, physical activity, food environment, or other individual and contextual factors not measured in the RNPT.

From a public health perspective, these findings highlight the value of repeated and individually linked anthropometric measurements for monitoring childhood nutritional status. Previous analyses of the RNPT have documented changes in the prevalence of overweight and obesity among Mexican schoolchildren between the 2015–2016 and 2018–2019 school years [10]. The present study extends this evidence by linking four consecutive measurements from the same children, thereby allowing heterogeneous longitudinal BMI-for-age patterns to be characterized, rather than relying exclusively on comparisons of population-level prevalence across measurement periods. Cross-sectional prevalence estimates are essential for nutritional surveillance, whereas longitudinally linked data provide complementary information on the persistence and evolution of BMI-for-age patterns during school years. The observed differences across trajectory groups also suggest that repeated anthropometric surveillance may help characterize population groups and contexts in which different longitudinal BMI-for-age patterns are more frequently observed. Nevertheless, the trajectory groups identified here are statistical representations of longitudinal patterns and should not be interpreted as deterministic categories or tools for predicting an individual child’s future weight status.

This study has several strengths. First, the large analytical sample included 463,244 children with four valid anthropometric measurements obtained over four consecutive school years, allowing BMI-for-age patterns to be examined longitudinally within the same children rather than through repeated cross-sectional comparisons. Individual records were linked across measurement periods using an encrypted and anonymized CURP, thereby enabling longitudinal follow-up while preserving confidentiality. Second, nutritional status was assessed using BMI-for-age z-scores based on the WHO 2007 growth reference, ensuring a standardized classification across the four measurement periods. Third, the large number of participating schools and the inclusion of contextual characteristics, such as school type, urban–rural setting, and municipal marginalization, allowed BMI-for-age patterns to be examined across different school and geographic contexts. Finally, the complementary use of latent class mixed models and population-averaged GEE models allowed us to characterize heterogeneity in longitudinal BMI-for-age z-score patterns and to examine changes in overweight/obesity across school grades in relation to individual and contextual characteristics.

Several limitations should be considered when interpreting these findings. First, the analytical cohort was restricted to children with one valid anthropometric measurement across all four consecutive school years. Consequently, children without complete longitudinal follow-up were not included, and potential differences between children with complete and incomplete measurements should be considered when interpreting these findings. Second, although the RNPT is a nationwide school-based nutritional surveillance system and the present analysis included many children and schools, the findings correspond specifically to children with four consecutive valid measurements and should not be extrapolated beyond the population represented in the analytical cohort. Third, the available data included a limited set of individual-level covariates and did not allow us to examine other factors potentially associated with BMI trajectories, such as dietary intake, physical activity, parental BMI, household socioeconomic characteristics, or pubertal development. Fourth, the urban–rural setting and municipal marginalization are contextual characteristics of the areas in which schools are located and should not be interpreted as measures of individual socioeconomic position. Finally, latent trajectory groups are model-derived representations of heterogeneous longitudinal BMI-for-age z-score patterns and should not be regarded as fixed clinical categories or deterministic developmental pathways.

## 5. Conclusions

Overall, this study provides longitudinal evidence of heterogeneous BMI-for-age z-score patterns among Mexican school-aged children followed across four consecutive school years. The identification of distinct trajectories, together with differences in the evolution of overweight/obesity across school grades and according to individual and contextual characteristics, illustrates the additional information obtained by linking repeated anthropometric measurements at the individual level. These findings reinforce the value of longitudinal anthropometric surveillance as a complement to cross-sectional prevalence estimates for characterizing changes in childhood nutritional status. Future studies incorporating individual, household, behavioral, and environmental characteristics are needed to better characterize the factors associated with these trajectories and to examine the mechanisms underlying the observed differences across population groups and contexts.

## Figures and Tables

**Table 1:** Baseline sample characteristics.

Characteristics	School Year	
	2015–2016	
	*n*	%
**Sex**		
Boys	233,790	50.5
Girls	229,454	49.5
**Age (yo)**		
5	5850	1.3
6	114,435	24.7
7	150,575	32.5
8	147,645	31.9
9	42,963	9.3
10	1776	0.4
11	0	0.0
12	0	0.0
**Grade**		
First	152,296	32.9
Second	154,457	33.3
Third	154,298	33.3
Fourth	2189	0.5
Fifth	3	0.0
Sixth	1	0.0
**School type**		
Indigenous or CONAFE	8479	1.8
Public	405,785	87.6
Private	48,980	10.6
**School municipal marginalization degree**		
Very high	4686	1.0
High	14,185	3.1
Medium	21,420	4.6
Low	30,426	6.6
Very low	392,527	84.7
**School setting**		
Rural	60,513	13.1
Urban	402,731	86.9
**Category of BMI/age z score**		
Thinness	18,200	3.9
Normal weight	287,119	62.0
Overweight	85,060	18.4
Obesity	72,865	15.7

**Table 2:** Distribution of trajectory group membership by sociodemographic characteristics (%).

	Low BAZ Pr (CI 95%)	Mid-Range BAZ Pr (CI 95%)	Elevated BAZ Pr (CI 95%)	Very-High BAZ Pr (CI 95%)
Sex				
Boys	12.3 (12.2, 12.5)	41.7 (41.5, 41.9)	32.0 (31.8, 32.1)	14.0 (13.9, 14.2)
Girls	13.8 (13.7, 14.0)	45.7 (45.5, 45.9)	32.1 (31.9, 32.2)	8.4 (8.3, 8.5)
School type				
Indigenous or CONAFE	15.6 (14.8, 16.4)	58.7 (57.7, 59.8)	23.0 (22.1, 23.9)	2.7 (2.4, 3.1)
Public	13.2 (13.1, 13.3)	43.6 (43.5, 43.8)	31.9 (31.8, 32.1)	11.3 (11.2, 11.4)
Private	12.0 (11.7, 12.2)	41.6 (41.1, 42.0)	34.3 (33.9, 34.7)	12.2 (11.9, 12.5)
School setting				
Rural	14.7 (14.4, 15.0)	49.6 (49.2, 50.0)	27.8 (27.4, 28.1)	7.9 (7.6, 8.1)
Urban	12.8 (12.7, 12.9)	42.8 (42.6, 42.9)	32.6 (32.5, 32.8)	11.7 (11.6,11.8)
School municipal marginalization degree				
Very high	14.5 (13.5, 15.5)	61.6 (60.3, 63.0)	20.7 (19.5, 21.8)	3.1 (2.6, 3.6)
High	15.8 (15.2, 16.4)	52.3 (51.4, 53.1)	25.8 (25.1, 26.5)	6.2 (5.8, 6.5)
Medium	14.2 (13.7, 14.7)	49.2 (48.5, 49.8)	28.3 (27.7, 28.9)	8.3 (8.0, 8.7)
Low	13.6 (13.2, 14.0)	45.7 (45.1, 46.2)	30.5 (30.0, 31.0)	10.3 (9.9, 10.6)
Very low	12.9 (12.8, 13.0)	42.7 (42.5, 42.9)	32.7 (32.5, 32.8)	11.7 (11.6, 118)

**Table 3:** Population-averaged generalized estimating equation (GEE) model for overweight and obesity among school-aged children.

Overweight & Obesity	Odds Ratio	95% CI	Pr(>|W|)
(Intercept)	0.01	0.01, 0.01	<0.001
School Grade	0.97	0.94, 0.99	0.0072
Low BAZ group	1.00		
Mid-range BAZ group	67.72	61.67, 74.37	<0.001
Elevated BAZ group	5.02	4.57, 5.52	<0.001
Very-high BAZ group	8126.96	7054.13, 9362.95	<0.001
School Grade × Low BAZ group	1.00		
School Grade × Elevated BAZ group	1.33	1.3, 1.37	<0.001
School Grade × Mid-range BAZ group	1.22	1.19, 1.26	<0.001
School Grade ×Very-high BAZ group	0.89	0.86, 0.93	<0.001
Sex: Boys	1.00		
Sex: Girls	0.94	0.93, 0.95	<0.001
School type: Indigenous or CONAFE	1.00		
School type: public	1.03	0.99, 1.08	0.1319
School type: private	1.01	0.96, 1.05	0.7713
Marginalization: very high	1.00		
Marginalization: high	1.1	1.04, 1.16	0.0012
Marginalization: medium	1.15	1.09, 1.21	<0.001
Marginalization: low	1.16	1.09, 1.22	<0.001
Marginalization: very low	1.29	1.22, 1.36	<0.001
School setting: Rural	1.00		
School setting: Urban	1.1	1.08, 1.12	<0.001

## Data Availability

The datasets presented in this article are not publicly available because they belong to the Salvador Zubirán National Institute of Medical Sciences and Nutrition. However, a de-identified public version of the datasets may be requested from the first author: Dr. Abelardo Cesar Ávila Curiel, e-mail: abelardoavila@yahoo.com.

## Supplementary Materials

The following supporting information can be downloaded at: https://www.mdpi.com/article/10.3390/nu18213532/s1. Supplementary Table S1. Decision criteria. Supplementary Material S1. Syntax model.

## Abbreviations

The following abbreviations are used in this manuscript:

RNPT	Registro Nacional de Peso y talla
Hlme	Linear Mixed-Effects
Lcmm	Latent Class Mixed Models
