# Supplementary material for: Longitudinal BMI-for-Age Trajectories Among Mexican School-Aged Children, 2015–2019
Source: Nutrients. 2026 Oct 27;18(21):3532. doi: 10.3390/nu18213532 (PMC13765328; doi:10.3390/nu18213532)
Supplement: Supplementary file 1 [file nutrients-18-03532-s001.zip › Supplementary Material S1. Syntax model.pdf]

Supplementary material.

S1. Model syntax

```
# Libraries required

library(haven)
library(arrow)
library(dplyr)
library(tidyr)
library(gt)
library(lcmm)
library(ggplot2)
library(scales)
library(geepack)
library(marginaleffects)
library(car)
library(carData)
library(nnet)
library(broom)



##### Data and coding

df_p <- read_parquet("BASE_ARTICULO_SIVNE_PRESERVED.parquet")

df_p$Sexo <- factor(df_p$Sexo,levels = c(1,2), labels=c('Hombres','Mujer'))
df_p$GM_2020_numerico <- factor(df_p$GM_2020_numerico,levels = c(1,2,3,4,5), labels=c('Muy
alto','Alto', 'Medio','Bajo', 'Muy bajo'))
df_p$EXT_CCT <- factor(df_p$EXT_CCT,levels = c(1,2,3), labels=c('E. indígenas','E. públicas', 'E.
privadas'))

GB <- subset(df_p, CICLO==2015, select = c(idesc, Grado))
colnames(GB)<- c("idesc", "GradoB")

df_p <- merge(df_p, GB, by = "idesc", all.x = TRUE)

df_model <- df_p %>%
  filter(CICLO %in% c(2015, 2016, 2017, 2018)) %>%
  mutate(time = CICLO - 2015)


##### Quadractic GBTM Models

### 1 group model
```

```
set.seed(961)
m1 <- hlme(imcz ~ time + I(time^2),
        subject = "idesc",
        data = df_model,
        ng = 1)


### 2 group model

# Note: Función gridsearch() is recomended to test several random starting values to matimize log-likelihood, we used fixed starting values due to computational limitations
# Recommended syntaxis is:

# m2 <- gridsearch(rep = 100, maxiter = 30, minit = m1,
#    hlme(imcz ~ time + I(time^2),
#        mixture = ~ time + I(time^2),
#        subject = "idesc",
#        data = df_model,
#        ng = 2))

# Used syntaxis is:
m2 <- hlme(imcz ~ time + I(time^2),
        mixture = ~ time + I(time^2),
        classmb = ~ GradoB,
        subject = "idesc",
        data = df_model,
        ng = 2,
        B = m1)


### 3 group model


m3 <- hlme(imcz ~ time + I(time^2),
        mixture = ~ time + I(time^2),
        classmb = ~ GradoB,
        subject = "idesc",
        data = df_model,
        ng = 3,
        B = m1)


### 4 groupo model

m4 <- hlme(imcz ~ time + I(time^2),
        mixture = ~ time + I(time^2),
        classmb = ~ GradoB,
```

```
         subject = "idesc",
         data = df_model,
         ng = 4,
         B = m1)


### 5 group model

m5 <- hlme(imcz ~ time + I(time^2),
         mixture = ~ time + I(time^2),
         classmb = ~ GradoB,
         subject = "idesc",
         data = df_model,
         ng = 5,
         B = m1)


### 6 group model

m6 <- hlme(imcz ~ time + I(time^2),
         mixture = ~ time + I(time^2),
         classmb = ~ GradoB,
         subject = "idesc",
         data = df_model,
         ng = 6,
         B = m1)


### 7 group model

m7 <- hlme(imcz ~ time + I(time^2),
         mixture = ~ time + I(time^2),
         classmb = ~ GradoB,
         subject = "idesc",
         data = df_model,
         ng = 7,
         B = m1)


## Model comparison


summarytable(m1, m2, m3, m4, m5, m6, m7,
           which = c("G", "loglik", "npm", "BIC", "%class"))

models <- list(m1, m2, m3, m4)

rel_entropy <- function(m) {
```

```
  ng <- length(unique(m$pprob$class))
  if (ng == 1) return(NA)
  probcols <- grep("^prob", names(m$pprob), value = TRUE)
  pm <- as.matrix(m$pprob[, probcols])
  1 - sum(-pm * log(pm), na.rm = TRUE) / (nrow(pm) * log(ng))
}

appa_by_class <- function(m) {
  ng <- length(unique(m$pprob$class))
  if (ng == 1) return(NA)
  probcols <- grep("^prob", names(m$pprob), value = TRUE)
  sapply(1:ng, function(k) mean(m$pprob[m$pprob$class == k, probcols[k]]))
}

occ_by_class <- function(m) {
  appa <- appa_by_class(m)
  if (all(is.na(appa))) return(NA)
  prop <- as.numeric(table(m$pprob$class) / nrow(m$pprob))
  (appa / (1 - appa)) / (prop / (1 - prop))
}

comp_tab <- data.frame(
  ng          = sapply(models, function(m) length(unique(m$pprob$class))),
  BIC         = sapply(models, function(m) m$BIC),
  entropy     = sapply(models, rel_entropy),
  min_class = sapply(models, function(m) min(table(m$pprob$class) / nrow(m$pprob) * 100)),
  min_APPA     = sapply(models, function(m) min(appa_by_class(m), na.rm = TRUE)),
  min_OCC      = sapply(models, function(m) min(occ_by_class(m), na.rm = TRUE))
  )

comp_tab %>%
  gt() %>%
    opt_table_font(size = "small") %>%
    tab_options(
      data_row.padding = px(4),
      column_labels.padding = px(4)
      ) %>%
  tab_header(title = "Selection criteria") %>%
      cols_label(
    ng       = "Group",
    BIC      = "BIC",
    entropy   = "Entropy",
    min_class = "min class %",
    min_APPA  = "min APPA",
    min_OCC   = "min OCC")


# Add class4 to data frame
```

```
class4 <- m4$pprob[, c("idesc", "class")]
colnames(class4)<- c("idesc", "class4")

class4$class4 <- factor(class4$class4)

df_model <- merge(df_model, class4, by = "idesc", all.x = TRUE)



# 4 group GBTM predicted BAZ

tim <- sort(unique(df_model$time))
newdata <- data.frame(
  time   = tim,
  GradoB = unique(df_model$GradoB)[1]
)

pred <- predictY(m4, newdata, var.time = "time")



# Plot for 4 group GBTM predicted BAZ

ggplot(df_pred, aes(x = time, y = y, group = class, color = class)) +
  geom_line(linewidth = 1) +
  geom_point(size = 2) +
  scale_color_discrete(
    labels = c("low BAZ", "elevated BAZ", "mid-range BAZ", "very-high BAZ")
  ) +
  labs(x = "Time", y = "Predicted standarized body mass index", color = "Group") +
  theme_minimal()



### Multinomial model for group characterization at baseline

mod1 <- multinom(class4~Sexo+EXT_CCT+GM_2020_numerico+AMBITO_2020,
data=subset(df_model, CICLO==2015), trace=FALSE)
Anova(mod2, type="III")

# Model coefficients
tidy(mod1, conf.int = TRUE, level=0.95, exponentiate = TRUE)

# Predicted probabilities for regressors

predictions(mod1, by = c("Sexo"))
```

```
predictions(mod1, by = c("EXT_CCT"))

predictions(mod1, by = c("AMBITO_2020"))

predictions(mod1, by = c("GM_2020_numerico"))



### Population-averaged model (GEE) to characterize differences in overweight and obesity across
school grades among the identified trajectory groups

# GEE model with ogit link function and binomial distribution family

mod2 <- geeglm(spyo~Grado*class4+Sexo+EXT_CCT+GM_2020_numerico+AMBITO_2020,
family=binomial(link="logit"), data=df_model, id=idesc, corstr = "exchangeable", std.err="san.se")

coef <- summary(mod2)$coefficients
coef <- tibble::rownames_to_column(coef, var = "term")

coef$OR <- exp(coef$Estimate)
coef$IC_inf <- exp(coef$Estimate - 1.96 * coef$`Std.err`)
coef$IC_sup <- exp(coef$Estimate + 1.96 * coef$`Std.err`)

pred <-predictions(
  mod2,
  newdata = datagrid(
    class4 = unique(df_class$class4),
    Grado = unique(df_class$Grado),
    Sexo = unique(df_class$Sexo)[1],
    EXT_CCT = unique(df_class$EXT_CCT)[1],
    GM_2020_numerico = unique(df_class$GM_2020_numerico)[1],
    AMBITO_2020 = unique(df_class$AMBITO_2020)[1]
))

ggplot(pred, aes(x = Grado, y = estimate, color = class4, group = class4)) +
  geom_line(position = position_dodge(width = 0.2), linewidth = 1) +
  geom_point(position = position_dodge(width = 0.2), size = 2) +
  geom_errorbar(
    aes(ymin = conf.low, ymax = conf.high),
    position = position_dodge(width = 0.2),
    width = 0.1
  ) +
  scale_y_continuous(
    breaks = seq(0, 1, by = 0.1),
    labels = label_percent(accuracy = 1)
  ) +
  scale_color_discrete(
    labels = c("low BAZ", "elevated BAZ", "mid-range BAZ", "very-high BAZ") #
```

```
) +
labs(
  x = "Grade",
  y = "Prevalence of overweight and obesity",
  color = "Group"

    ) +
theme_minimal()
```
