# Supplementary material for: Longitudinal BMI-for-Age Trajectories Among Mexican School-Aged Children, 2015–2019
Source: Nutrients. 2026 Oct 27;18(21):3532. doi: 10.3390/nu18213532 (PMC13765328; doi:10.3390/nu18213532)
Supplement: Supplementary file 1 [file nutrients-18-03532-s001.zip › Supplementary Table S1. Decision criteria.pdf]

**Supplementary table S1. Decision criteria[¥]**

| Groups | BIC | Entropy | Min class % | Min APPA | Min OCC |
|---|---|---|---|---|---|
| 1 | 222211.6 | NA | 100.000000 | Inf | Inf |
| 2 | 201389.1 | 0.8100397 | 38.105372 | 0.9335218 | 11.875161 |
| 3 | 194525.7 | 0.7863966 | 22.763913 | 0.8889888 | 8.141995 |
| 4 | 192053.9 | 0.7768660 | 10.163152 | 0.8501557 | 8.034619 |
| 5 | 191088.1 | 0.7491435 | 4.211001* | 0.8074690 | 7.985032 |
| 6 | 190318.2 | 0.7714207 | 1.799187* | 0.7843949 | 8.389618 |

*Below the recommended threshold

¥ This table was generated using a 10% subsample of the subjects as a first step toward determining the number of groups. Subsequently, analyses were run for 2 to 4 groups to establish the classification for all included subjects.
